# Using the Community Readiness Model and Stakeholder Engagement to Assess a Health System’s Readiness to Provide LGBTQ+ Healthcare: A Pilot Study

**DOI:** 10.21203/rs.3.rs-1902727/v1

**Published:** 2023-03-29

**Authors:** Madelyne Z Greene, Molly M Herrmann, Bryce Trimberger

**Affiliations:** University of Wisconsin-Madison School of Nursing; Humble Pie Consulting, LLC; University of Wisconsin-Madison School of Nursing

**Keywords:** Health Equity, LGBT Health, Discrimination in Healthcare, Sexual and Gender Minorities

## Abstract

**Background:**

Despite broad social and policy changes over the past several decades, many LGBTQ+ people face barriers to healthcare and report mistreatment and disrespect in healthcare settings. Few health systems level interventions have been shown to improve sexuality- and gender-related health disparities. Using the Community Readiness Model, we developed and implemented a rigorous assessment and priority-setting intervention at one mid-sized health system in the midwestern US. We evaluated the system’s readiness to provide LGBTQ+ healthcare and developed immediate action steps that are responsive to local context. We engaged diverse stakeholder groups throughout the process.

**Methods:**

Led by the Community Readiness Model, we identified key groups within the health system and conductedstructured interviews with 4–6 key informants from each group. Two trained scorers external to the study team individually scored each interview on a numerical scale ranging from 1 (no awareness of the problem) to 9 (community ownership of the problem) and discussed and reconciled scores. Group scores were averaged for each dimension of readiness and overall readiness, and then triangulated with stakeholders to ensure they reflected lived experiences. Finally, specific recommendations were generated to match the needs of the system and move them towards higher levels of readiness.

**Results:**

We convened an advisory committee of LGBTQ+ patients of the health system and a panel of local experts on LGBTQ+ wellness. Both groups contributed significantly to research processes. 28 interviews across 6 staff subcommunities indicated readiness levels ranging from “3: Vague Awareness” of the issue, and the “4: Preplanning” stage. Discrepancies across staff groups and dimensions of readiness suggested areas of focus for the health system. The evaluation process led to immediately actionable recommendations for the health system.

**Conclusions:**

This pilot study demonstrates the potential impact of the Community Readiness Model on improving health systems’ readiness to provide LGBTQ+ healthcare. This model combines strengths from community-based research and implementation science approaches to form an intervention that can be widely disseminated and maintain the flexibility and agility to meet local needs. Future research will evaluate changes in readiness at the same health system and test the process in additional health systems.

## Introduction

Nearly 9 million Americans identify as lesbian, gay, bisexual, transgender, queer, and/or other non-heterosexual or gender diverse identities (LGBTQ+) (1). As conceptualized by Bronfenbrenner’s Socio-Ecological Theory (2), the County Health Rankings Model (3), the Social Determinants of Health (4), and other prevalent conceptual theories, LGBTQ + individuals face barriers to health at multiple levels, including interpersonal, social and community contexts, access to education and employment opportunities, and—critically—health care access and quality. With a population of this size, virtually all healthcare providers will encounter numerous LGBTQ + individuals throughout their career. However, relatively few efforts are made to train health care providers on LGBTQ + care competencies. As a result, many LGBTQ + people continue to report barriers to care and mistreatment and disrespect in health care settings. Additionally, few studies have developed and rigorously tested interventions in clinical care settings that aim to address sexuality- and gender-related health disparities.

Decades of research have consistently shown that LGBTQ + people bear disproportionate burdens of many mental and physical health concerns across the lifespan (5–8). Disparities not only exist between LGBTQ + individuals and the heterosexual and cisgender population, but also *within* the LGBTQ + community. Estimates show that nearly 40% of transgender adults attempt suicide in their lifetime (9), compared to 17% among lesbian, gay, and bisexual adults (10), and only 0.6% in the general population (11). Transgender women of color face interpersonal and hate-related violence at much higher rates than any other group with LGBTQ + identities (12, 13). The “LGBTQ + community” is a large and diverse population with as many differences as ties. The scientific and policy communities have paid some increasing attention to the presence of LGBTQ + health disparities over the past several decades. For example, the Healthy People 2020 and 2030 Initiatives include objectives related to LGBTQ + health, including reducing adolescent bullying, preventing and treating substance use, reducing mental health burden, preventing sexually transmitted infections including human immunodeficiency virus (HIV), and increasing data collection on LGTBQ + populations (14, 15).

Disparities related to sexuality and gender identity can also be compounded by additional levels of discrimination such as racism, socioeconomic injustice, and disability. Broad social and policy changes such as the legalization of same-sex marriage in 2015 were predicted to reduce some of these barriers by expanding access to insurance coverage for LGBTQ + families, but disparities have persisted (16) and broad legislative changes cannot be seen as simple remedies for the complex causes of health disparities among LGBTQ + people (17).

In addition to the broader socio-structural causes of health inequities among LGBTQ + people, barriers to high quality healthcare remain. LGBTQ + people commonly report experiences of discrimination and gatekeeping in healthcare settings (18–21). Most transgender individuals have encountered harassment, gatekeeping, misgendering or deadnaming (using a former or “birth” name after a person has transitioned and changed their name), belittlement or ridicule, or denial of care (18, 22, 23). One-third of gay and bisexual men and half of lesbian and bisexual women report negative healthcare experiences in the past year. Among lesbian, gay, bisexual, and queer youth and adults, an estimated 37% avoid disclosure of sexual identity to healthcare providers altogether (18, 24). These experiences of, and even fear or expectation of discrimination, lead to missed and delayed care, further contributing to poor health outcomes (18, 25–28).

Many providers continue to be unaware of the health needs and concerns of LGBTQ + patients, and often lack the training or even vocabulary that they need to provide high-quality care. There is a widely documented lack of content on LGBTQ + populations and health in medical, nursing, and other health professional training (19, 23, 29–37). Clinicians may avoid questions related to sexual orientation and gender identity or assume that their patients are heterosexual and cisgender as a result of their own discomfort or ignorance (38). This can further contribute to difficult encounters and avoidance of care (23). Some previous research has shown that cultural competency and nondiscrimination training does not adequately address these training gaps (7, 39).

LGBTQ + populations have a rich history of creating community-based support and systems that address healthcare needs as a response to the neglect and mistreatment of the formal healthcare system. These networks and resources persist today; LGBTQ + communities often share information about which providers are safe and competent in caring for LGBTQ + patients and which should be avoided (40). However, these networks do not adequately replace the need for access to affirming and competent healthcare from health professionals. There are also several clinics across the country that specialize in providing primary and preventative care to LGBTQ + people. However, they are concentrated in specific large urban areas and do not often provide specialty care.

While these disparities and challenges have been well documented, very few studies have developed and tested interventions aimed to improve LGBTQ + healthcare and outcomes, and none have used an implementation science approach to develop a scalable intervention that meets the scope of these issues. This study therefore uses the Community Readiness Model (CRM) to pilot test a health systems intervention involving deep reflection and data gathering about current LGBTQ + health care delivery. The CRM measures an organization or system’s “readiness” for a particular action; in this case, providing high-quality LGBTQ + healthcare. A system-level intervention informed by implementation science, grounded in change theory, and implemented with community and stakeholder engagement has the potential to identify opportunities for measurable change and increase health systems’ capacity to provide high-quality care to LGBTQ + patients.

## Study Aims

Given the evidence about LGBTQ + persons’ experiences in healthcare settings and the complexity of inducing effective organizational change, we aimed to accomplish two major tasks. First, we aimed to engage diverse stakeholders to inform and reflect on the Community Readiness Assessment process and its utility for generating increased readiness to provide high quality LGBTQ + healthcare. Second, we aimed to develop and complete a pilot implementation of the Community Readiness Assessment process to assess one mid-sized health systems’ readiness for LGBTQ + healthcare and generate an evidence-based action plan for improvement.

## Methods

### The Community Readiness Model (CRM)

The Community Readiness Model (CRM) guided our study design and process. The CRM was originally designed to guide communities towards increased readiness for change and has since been applied to formal entities such as healthcare organizations. The CRM is based on the Transtheoretical Model of Behavior Change (41) and incorporates principles of implementation science icluding iteration and adaptation to local context. It helps assess the “readiness” of a group or organization for change in a desired area and then makes specific recommendations for next steps based on the assessed readiness level. The model measures 5 key dimensions of readiness: knowledge about the issue of interest, knowledge of already existing local efforts and work on that issue, community climate, leadership engagement, and what resources are invested in addressing the issue (Table 1). Readiness is measured in 9 distinct stages, ranging from “no awareness of the problem” (stage 1) to “community ownership of the problem” (stage 9). The stages of readiness represent the level of knowledge, resources, and efforts in place to address the issue of interest (42). Procedures for evaluating readiness and generating recommended action steps are detailed in the CRM Handbook (43).

In this case, the “community” being assessed is the health system, distinct from the LGBTQ+ community impacted by the issue. Informed by stakeholder-informed research methods, we aimed to recruit and engage two LGBTQ+ advisory groups to evaluate and advise on the entire process of sampling, data collection, and dissemination of findings. Details about the resulting composition and functions of these stakeholder groups are reported as results of our first aim below.

### Study Procedures

We first identified the community targeted for change. For this pilot study, we selected a local mid-sized health system consisting of several outpatient clinics offering primary, preventative, urgent, and some specialty care. This system was selected because they are community-oriented and have expressed interest in LGBTQ+ healthcare by participating in various local efforts and events, but do not have a particular focus on or expertise in LGBTQ+ health. Following the steps outlined in the handbook and with the input of the PAG, we then selected subcommunities, or specific groups within the health system, to target for interviews. Subcommunities were selected with the input of the stakeholder groups because of their direct interaction with patients and ability to enact change in the system. They included reception team members, clinical support staff such as medical and nursing assistants, primary care providers, behavioral health providers, member services staff who assist patients with insurance coverage and payment issues, and directors and senior leadership.

We then conducted structured CRM interviews via telephone with between 4 and 6 key informants from each subcommunity, as recommended by the CRM (42). Interviews lasted between 30 and 70 minutes. They were transcribed and then scored and interpreted by two CRM-trained scorers external to the study team. The two scorers’ individual scores were discussed and reconciled for each interview, and then average subcommunity scores were calculated for overall readiness and in each readiness dimension. The scores produced by this process were then triangulated with both stakeholder groups to ensure they were reflective of their lived experiences.

Finally, based on the readiness scores and the nuanced information about the health system that had been generated, specific action steps were recommended to move them towards higher levels of readiness. For example, according to the CRM, a community with a readiness score of 3 (vague awareness of the problem) might employ interventions such as educational sessions, informational flyers, and community events to help increase public awareness of the issue. Findings and recommended action steps were then collated and developed into a toolkit for use by the health system.

## Results

### Aim 1. Engage Stakeholders to Inform and Reflect on the Community Readiness Assessment

#### Patient Advisory Group (PAG)

Following recommended practices for stakeholder engagement, we assembled a patient advisory group (PAG) of current patients in the health system, with representation from individuals who were lesbian, gay, bisexual, queer, transgender, and gender nonbinary (Table 2). From a pool of approximately 40 individuals who reached out to us with interest in the PAG, we established a final group of 12 people. There were 7 white and 5 non-white members, and this relative lack of racial and ethnic diversity was discussed regularly by PAG members and the research team. The PAG did represent significant diversity in age and services utilized within the health system.

The purpose of the PAG was to incorporate the perspectives of actual patients in the planning and execution of the study. Members of the PAG met six times over the course of the study and successfully achieved five main tasks: 1) consulting on the CRM interview guide, including specific language to name the “problem” of LGBTQ+ health and healthcare; 2) identifying subcommunities of staff members to interview with a focus on staff roles that had the biggest impact on their care experiences; 3) sharing stories of both positive and negative experiences within the organization to illustrate findings; 4) vetting and interpreting results from the interviews; and 5) advising on next research steps.

Initial interest in the PAG was high, and we included more members than our originally planned eight-to-ten members to maximize diversity of both sexual and gender identities and race and ethnicity. However, retention in this group was moderate; seven of the twelve members attended at least five of the 6 planned meetings. An additional three members only attended the first three meetings and did not return after a larger time gap passed during the data collection phase. Our team’s attempts to collect information about why those members did not return for future meetings were unsuccessful. Individual feedback from those who attended after each individual meeting was generally positive.

#### Professional Advocate Team (PAT)

We also successfully established a Professional Advocate Team (PAT), comprised of 6 local leaders in LGBTQ+ health, healthcare, and equity. Members represented organizations that support LGBTQ+ community building, Black and Southeast Asian LGBTQ+ communities, transgender health activism, and LGBTQ+ inclusion in schools. They were identified and recruited through professional networking and individual outreach.

The PAT group met twice. At the beginning of the study processes, the PAT reviewed the CRM framework, our planned interview process, and the subcommunities selected by the PAG. This group reconvened after all data collection was complete to review our initial findings and preliminary interpretations of scored data, with a focus on institutional perspectives and change processes. Responses to the PAT meetings were positive and successful in eliciting detailed feedback and thoughtful advice related to strategies to promote institutional change. For example, the PAT’s input confirmed our decisions to include Directors and Senior Leadership as a subcommunity for data collection. The PAT’s feedback also improved the recommended action steps generated by the study team so that they truly targeted changes at the system level, not only at the level of individual staff members.

### Aim 2. Develop and Implement a Process to Assess Health Systems’ Readiness for LGBTQ+ Healthcare

We conducted 28 interviews with staff members across the 6 subcommunities identified by the PAG. Subcommunities were 1) members of the reception team; 2) clinical support staff including medical and nursing assistants; 3) member services staff, who assist patients with insurance coverage and payment issues; 4) primary care providers; 5) behavioral health care providers including psychologists, therapists, and psychiatrists; and 6) members of the senior leadership team of the health system. Table 3 displays the demographic characteristics of the study sample and Table 4 shows the overall and dimension-specific readiness levels of each subcommunity.

As shown in Table 4, subcommunities scored either a “3,” indicating “Vague Awareness,” or a “4,” indicting a “Preplanning” stage of readiness. Subcommunities that were assessed to be in the “Vague Awareness” stage of readiness were members of the reception team, clinical support staff, and member services staff. Subcommunities that were assessed to be in the “Preplanning” stage of readiness were primary care providers, behavioral health care providers, and members of the senior leadership team.

“Vague Awareness,” indicated by a score of 3, indicates that most people in the community believe that there are local concerns—suggesting that they would agree that LGBTQ+ communities experience some disparities or barriers to care—but there is no immediate motivation to actively address those concerns. At this stage, community members will have only vague knowledge about the issue with little specific information or experience. A few community members will have at least heard about the current local efforts towards LGBTQ+ health, but know little about them, and limited resources are available to address the issue. Members of communities at the “Vague Awareness” stage may find themselves asking, “Something should probably be done, but what? Maybe someone else will work on this.”

“Preplanning,” indicated by a mean readiness score of 4, indicates a slightly more advanced stage of readiness. This stage suggests that both community members and leadership acknowledge that the issue is a concern and agree that something must be done to address it. There is an increased sense of relevance and responsibility for the problem. However, at this level, community members will still have limited knowledge about LGBTQ+ health and health care, most community members will know little about current local efforts, and there will still be limited resources available to be used for further efforts to address the issue. The Preplanning stage may be represented by a sentiment like, “This is important. What can we do?”

As dictated by the CRM process, these scores were presented to both stakeholder groups. The PAG reviewed scores in each subcommunity and each dimension to evaluate whether it reflected their experience. There was overall validation of the calculated levels of readiness. Members of the PAG also discussed their own experiences in terms of the calculated scores; these stories were later used to illustrate findings in the Toolkit generated for the health system and its staff. The PAT also reviewed the generic, model-generated recommended action steps to assess their usefulness and applicability in the current organizational setting. The group’s feedback contributed to tailoring the recommended actions.

## Discussion

### Patient and Stakeholder Engagement

In this pilot study, we successfully completed the aims related to stakeholder and patient engagement. We recruited and sustained both an advisory group of LGBTQ+ patients of the health system (PAG) and a separate group of local experts in LGBTQ health and wellbeing (PAT). Both advisory groups served critical functions throughout the research process. The PAG did have a relatively low retention rate; this seems to corroborate pre-existing literature about stakeholder engagement which suggests that recruiting a smaller number of members may lead to more engagement among all members, stronger group cohesion, and ultimately better retention of group members.

### Subcommunity Readiness Scores

The readiness assessment process resulted in relatively consistent scores between subcommunities: ranging only between “3: Vague Awareness” and “4: Preplanning” stages. However, two important patterns in readiness scores emerged. First, there were meaningful differences between the subcommunities that were assessed at each stage of readiness. The three subcommunities that were assessed at a “Vague Awareness” stage included the groups of staff members that tend to have the least institutional power and influence. Clinical staff such as receptionists, who interact with patients but are often not considered health professionals, may have less formal training, and have much lower access to formal and continuing education opportunities where expansion of LGBTQ+ health knowledge often takes place (44–46). In contrast, the three subcommunities that were assessed at a slightly more advanced level of readiness— “Preplanning”—had significantly more institutional power and included both physicians and the health system’s management and senior leadership team.

Members of the PAG reflected on the importance of this discrepancy in their lived experience as patients in the health system. While all agreed that leadership engagement is critical to generate continuous change and improvement, the subcommunities with less institutional power are often the people who engage first or most often with patients themselves. If readiness to provide high quality care for LGBTQ+ patients does not extend to those staff members, then patients remain at risk of experiencing incompetent or disrespectful care. Consistent with the CRM, we recommended that the health system focus their first improvement efforts on reaching these subcommunities and balancing disparities in readiness across the health system.

### Dimensions of Readiness

Second, although the CRM suggests that users focus on readiness scores *within* each subcommunity, there were also notable patterns in readiness level *across* subcommunities in each of the 5 dimensions of readiness. The Leadership dimension was consistently scored as the highest or second highest dimension of readiness. Across all six subcommunities, the Leadership dimension had a mean readiness score of 4.9, reflecting aspects of “4: Preplanning” and “5: Preparation” stages. These levels suggest that at least some of the leadership believes that this issue is a concern in the community and that some type of effort is needed to address it. Some may be passively supportive of current efforts, some may be participating in developing, improving, or implementing efforts, but only a few members of leadership play any active roles in those efforts. This very accurately reflected the local system context. This relatively higher score in the Leadership dimension demonstrates that members of leadership had interest in or concern about LGBTQ+ health, and that those concerns had reached some other team members.

In contrast, the dimension “Knowledge of the Issue” consistently scored lowest or second lowest across all 6 subcommunities, with a mean score of 3.1. This reflects the “Vague Awareness” stage of readiness, suggesting that at least some community members have heard about LGBTQ+ health concerns, but lack detailed information about the issues or actions that could be taken to address them. There may be stereotypes or harmful misconceptions about LGBTQ+ health and healthcare in the community at this stage. This finding was particularly well supported by the experiences and feedback of the PAG members. They shared many instances when beginning efforts were made, including visual indicators of inclusion like posters or flags during June (LGBTQ Pride month or clinical staff asking about LGBTQ+ identity and making attempts to use inclusive language, but then not having the skills or information they needed to address related health concerns. In some cases, PAG members had been given incorrect medical guidance because of a lack of knowledge.

These disparities across dimensions of readiness were of concern to both PAG and PAT members. The PAT expert panel emphasized the potential damage that can be done when people in an organization take action on an issue while they still lack critical pieces of knowledge. In particular, this can result in missed screening and preventative care, disrespect stemming from ignorance about a particular population, and LGBTQ+ people feeling misled when they receive care from an organization that asserts their inclusivity but is not prepared to meet specific health needs.

### Recommended Action Steps for Enhancing Readiness

The CRM recommends specific action steps at each measured level of readiness, which were then tailored to the specific research site and triangulated with the priorities and experiences of the PAG and the expertise of the PAT members. 8–10 resulting recommendations for each subcommunity were generated and shared with the health system leadership various other groups within the health system. As discussed above, the model guides the organization to focus on lowest-scoring areas first, which included the reception, clinical support, and member services staff members and the “knowledge of the issue” and “resources” dimensions of readiness. Examples of actions steps in these areas are reported in Table 5. Our research team collaborated with a marketing firm that specializes in health communication to craft a Toolkit for the health system under study. The Toolkit summarizes our findings and the resulting recommended action steps in an easy-to-digest format and includes stories, or “testimonials” from PAG members to illustrate findings.

## Conclusions

This pilot implementation study demonstrated that our intervention, based on the Community Readiness Model, has the potential to create real change towards LGBTQ+ health equity at the health systems level. We had successful participation from multiple stakeholders, successfully recruited and interviewed staff at the healthcare system, and generated findings that informed actionable, locally grounded action steps for the health system to enhance their care of LGBTQ+ patients. The CRM method successfully balanced the adaptability and flexibility that will be needed to address the huge variety of “starting places” in LGBTQ+ care with the rigor and depth needed to reproduce impact. This balance illustrates the value of an implementation science approach to developing such interventions, particularly when combined with meaningful stakeholder engagement.

Having completed this successful pilot study, our immediate research plans include returning to the health system to re-assess readiness, measuring the impact of the process on both measured readiness and actual patient experiences. In order to make an impact on the readiness of health systems all over the country to provide high-quality care to LGBTQ+ patients, and ultimately improve the health of LGBTQ+ people, it will be critical to ensure that this approach has broad and consistent impact. Therefore, we also plan to expand this research to test the intervention and its impact in additional health systems.

## Figures and Tables

**Table 1.** Dimensions of organizational readiness for change according to the Community Readiness Model (CRM).

Dimension	Evaluative Question
Community Knowledge of the Issue	How much does the community know about the issue, its causes, and impacts?
Community Knowledge of Efforts	How much does the community know about current programs and activities related to the issue?
Community Climate	What is the community’s attitude toward addressing the issue?
Leadership	What is leadership’s attitude toward and involvement in addressing the issue?
Resources	What are the resources that are already being used or could be used to address the issue?

**Table 2.** Demographic Characteristics of the Patient Advisory Group (PAG) (N=12)

**Age**	
Mean	32 years
Range	21–50 years
**Race/Ethnicity**	
White	7
Asian, inc. mixed race white and Asian	3
Latinx	2
**Health Services Utilized**	
Primary Care	12
Behavioral Health	6
Urgent Care	4
Pediatrics (as parent)	2

**Table 3.** Demographic Characteristics of the Study sample of staff (N=28)

**Age (years)**	
Mean	45.6
Range	31–67
**TIME at HEALTH SYSTEM (YEARS)**	
Mean	6.1
Range	0.5–37
**Gender**	
Female/Woman	21
Male/Man	5
Gender Nonbinary	2
**Staff role**	
Reception Team	4
Clinical Support Staff	4
Member Services	4
Primary Care Providers	6
Behavioral Health Providers	6
Directors and Senior Leaders	4

**TABLE 4.** READINESS TO PROVIDE LGBTQ+ HEALTHCARE IN A MID-SIZED OUTPATIENT HEALTH SYSTEM, BASED ON COMMUNITY READINESS MODEL SCORING.

Readiness Dimension	Average Scores Across 4–6 staff members					
	Reception Team	Clinical Support	Primary Care Providers	Behavioral Health Providers	Member Services	Directors & Senior Leaders
Knowledge of Efforts	3	2.5	4.83	5.5	3.96	5.5
Leadership	4	4.75	5.75	5	4.88	5
Community Climate	4	3.75	4.83	4.33	4.3	4.63
Knowledge of Issue	2.75	2.5	3.67	3.17	3.02	3.75
Resources	3.5	3	3.33	3.5	3.33	4.25
Mean	3.45	3.3	4.48	4.3	3.88	4.63
Readiness score	**3**	**3**	**4**	**4**	**3**	**4**

**Table 5.** Example recommendED action steps in priority areas generated by the Community Readiness Model and tailored to the health system under study.

Targeted Subcommunities	Dimension of Readiness	Recommended Action
Reception Team Clinical Support Staff Member Services	Knowledge of Efforts	Ensure that all organization-wide efforts are scaled and accessible to all staff.
	Knowledge of the Issue	Actively promote and consider requiring LGBTQ+ health training modules for all staff.
		Recruit staff in these roles to the existing “LGBT health committee” in the health system.
All		Collect specific data about patient interactions through surveys, focus groups, or a patient advisory group, and share those patient stories with staff.
	Resources	Develop budgetary plans and commitments for LGBTQ+ education, learning opportunities, and community outreach.
		Incentivize training events, being sure that incentives are equivalent across employee communities.

## Data Availability

The datasets generated and/or analyzed during the current study are not publicly available due to the potential for identifying the specific organization under study but are available from the corresponding author on reasonable request. Group-level data generated and analyzed during this study are included in this published article.

## Abbreviations

LGBTQ+: lesbian, gay, bisexual, transgender, queer, and/or other non-heterosexual or gender diverse identities
LGBT: lesbian, gay, bisexual, transgender (used when in reference to other work or groups that utilize this acronym)
HIV: human immunodeficiency virus
CRM: Community Readiness Model
PAG: Patient Advisory Group
PAT: Professional Advocate Team
